# Roles of Heme Oxygenase-1 in Neuroinflammation and Brain Disorders

**DOI:** 10.3390/antiox11050923

**Published:** 2022-05-08

**Authors:** Yi-Hsuan Wu, Hsi-Lung Hsieh

**Affiliations:** 1 Research Center for Chinese Herbal Medicine, College of Human Ecology, Chang Gung University of Science and Technology, Taoyuan 333, Taiwan; yhwu03@mail.cgust.edu.tw; 2 Department of Nursing, Division of Basic Medical Sciences, Graduate Institute of Health Industry Technology, Chang Gung University of Science and Technology, Taoyuan 333, Taiwan; 3 Department of Neurology, Chang Gung Memorial Hospital, Taoyuan 333, Taiwan

**Keywords:** heme oxygenase, neuroinflammation, neurodegenerative diseases, Alzheimer’s disease, Parkinson’s disease

## Abstract

The heme oxygenase (HO) system is believed to be a crucial mechanism for the nervous system under stress conditions. HO degrades heme to carbon monoxide, iron, and biliverdin. These heme degradation products are involved in modulating cellular redox homeostasis. The first identified isoform of the HO system, HO-1, is an inducible protein that is highly expressed in peripheral organs and barely detectable in the brain under normal conditions, whereas HO-2 is a constitutive protein that is highly expressed in the brain. Several lines of evidence indicate that HO-1 dysregulation is associated with brain inflammation and neurodegeneration, including Parkinson’s and Alzheimer’s diseases. In this review, we summarize the essential roles that the HO system plays in ensuring brain health and the molecular mechanism through which HO-1 dysfunction leads to neurodegenerative diseases and disruption of nervous system homeostasis. We also provide a summary of the herbal medicines involved in the regulation of HO-1 expression and explore the current situation regarding herbal remedies and brain disorders.

## 1. Introduction

Heme oxygenase (HO) is an evolutionarily conserved enzyme and is involved in many different diseases. HO plays the role of a rate-limiting enzyme in degrading endogenous iron protoporphyrin heme by release of carbon monoxide (CO), biliverdin (BV), and ferrous ions (Fe^2+^), which could be recycled for heme homeostasis.



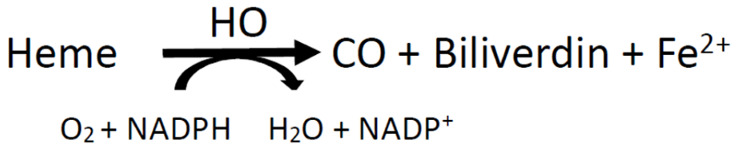



**Figure 1:**
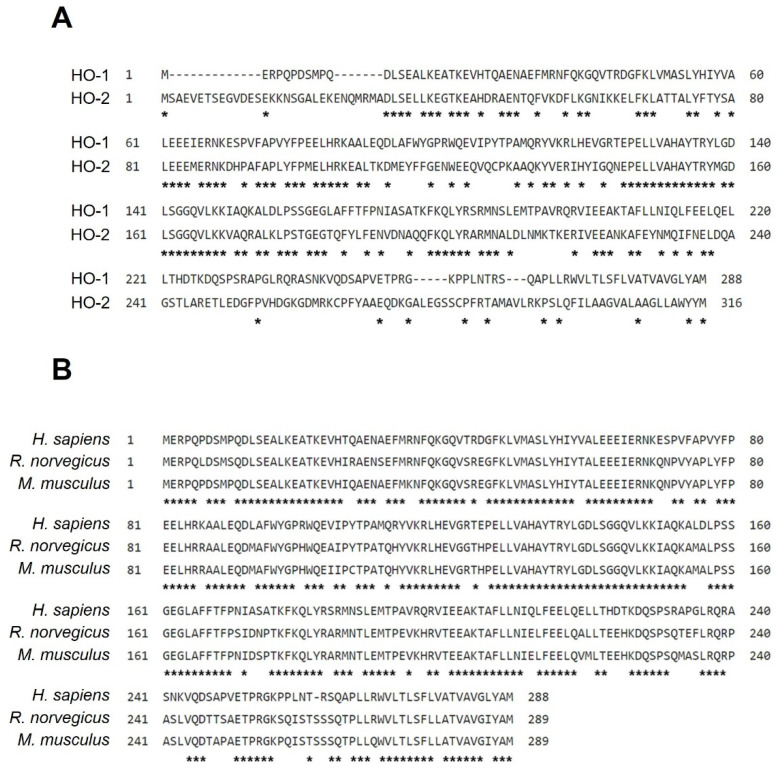
The amino acid alignment of HO. (**A**) Amino acid alignment of HO-1 and HO-2 in human. (**B**) The amino acid homology of HO-1 in humans, rat, and mice. Asterisks indicate common retention regions, meaning that the amino acids here are identical.

There are three isoforms of the HO system: HO-1, HO-2, and HO-3. Interestingly, HO-3, a pseudogene discovered in rat, is a splice-variant of HO-2 and remains elusive and poorly understood [1,2]. The amino acid alignments of HO-1 and HO-2 are shown in Figure 1A; they demonstrate a 43% homology the amino acid sequence of humans. HO-1, encoded by a gene called *HMOX1*, is a well-known inducible isoform and can be transcriptionally upregulated as much as 100-fold as a result of stimuli, such as radiation, toxins, infections, and injuries [3]. HO-2, encoded by a gene called *HMOX2*, is a constitutively expressed protein and is present in high levels in the brain [4].

HO is the rate-limiting enzyme of heme degradation, and the end-products, which include CO, Fe^2+^, and BV (converted into bilirubin (BR) by biliverdin reductase), play important roles in regulating cellular homeostasis. BR is more electrophilic than BV and thereby comparatively increases the reactivity of Kelch-like erythroid cell-derived protein with CNC homology-associated protein 1 (Keap1) to release Nrf2 [5]. The Keap1–Nrf2 system has been well studied in mammalian cells, especially its protection role against oxidative stress in organisms. CO is well-known for its antioxidant, vasodilator, anti-inflammatory, and anti-apoptotic effects, among others. Therefore, HO and its heme degradation products are potent protective modulators under oxidative stress conditions.

The controversial role of HO-1 is explored in several studies, e.g., they both delineate the importance of its antioxidant activity and also demonstrate its function in the development of diseases. In this review, we summarize the essential roles of HO-1 and its end-products for ensuring brain health and further discuss how HO-1 dysfunction leads to several neural disorders, such as Parkinson’s disease (PD) and Alzheimer’s disease (AD). We also review the ongoing clinical herbal trials aimed at exploring the therapeutic targets derived from HO-1 regulation for the treatment of neural disorders.

## 2. The Functions of HO-1 in Brain Physiology

### 2.1. Overview

The brain is the most important organ in the human body and requires sufficient oxygen to maintain its functions, i.e., it needs to consume 20% of the total basal oxygen to support intensive neuronal activity [6]. As a result of the transport and storage of oxygen, heme is necessary for the survival of most organisms. Moreover, in the central nervous system (CNS), redox homeostasis is involved in development, aging, and neural diseases [7]. Since HO is the rate-limiting enzyme in heme degradation and can be modulated by redox status, the role of the HO system is important for maintaining brain function. Current studies demonstrate that dysregulation of the HO system is associated with the pathogenesis of neurodegenerative diseases, such as AD, PD, and multiple sclerosis (MS) [8,9], and is even involved in neurotoxicity and neuroinflammation.

HO-1 was first identified in 1968, and many studies focused on the regulation and function of this protein in heme metabolism [10]. There is a high amino acid homology of HO-1 in humans, mice, and rats (Figure 1B). However, increasingly, amounts of evidence over in recent decades demonstrate that HO-1 could be induced by a variety of inducers other than heme [11,12], such as heat shock, heavy metals, endotoxin, inflammatory cytokines, and even oxidative stress, indicating that HO-1 plays a vital role in modulating cellular homeostasis. Interestingly, HO-1 induction with increased heme degradation products confers antiviral activity by interferon activation against a wide range of viruses, such as HIV, influenza, respiratory syncytial virus, enterovirus 71, human herpes simplex virus, and respiratory syndrome virus, etc. [3]. A current study also indicates that HO-1 activation may be a possible therapeutic strategy against COVID-19-associated complications [3,13]. All these investigations indicate that HO-1 plays a vital role in regulating human physiopathology.

### 2.2. The Canonical and Non-Canonical Effects of HO-1 in Brain

The by-products of heme degradation by HO-1 include BV, CO, and Fe^2+^, and the canonical effects of HO in the brain include antioxidant, anti-apoptosis, vasodilation, and anti-inflammatory responses [14,15,16,17]. Due to the direct antioxidant property [18], BV administration in rats can ameliorate damage to the brain by reducing oxidative DNA damage [19]. Furthermore, BV alleviates pro-inflammatory responses through the NF-κB pathway [20] and inhibits toll-like receptor 4 (TLR4) signaling [21], which is the main contributor to neurological disorders [22,23]. Moreover, CO in the brain is an activator of guanylyl cyclase and functions as a neurotransmitter [24,25]. Astrocytic mitochondrial biogenesis can be stimulated by CO through L-type Ca^2+^ channel-mediated PGC-1α/ERRα signaling [26]. Although it does not directly influence the brain tissue, CO exhibits anti-apoptosis and anti-inflammatory effects in the lungs of brain-dead rats through p38-MAPK signaling [27]. CORM-A1 supplements, i.e., a carbon monoxide donor, offer a novel and effective therapeutic agent against cerebrovascular dysfunction caused by neonatal seizures [28] and experimental allergic encephalomyelitis [29].

Interestingly, aside from the canonical effect, recent studies demonstrate that HO-1 also possesses other physiological functions, which are not correlated with their own enzymatic functions; these are termed “non-canonical functions”. Those non-canonical functions contain protein–protein interaction, intracellular compartmentalization, and extracellular secretion [30]. The protein–protein interaction of HO isoforms was first observed in 1977 [31]. An interaction between HO-1 and HO-2 proteins serves to limit HO activity [32], indicating a possible cytoprotective range of HO expression in brain tissues.

The second non-canonical effect of HO-1 is intracellular compartmentalization. Although studies demonstrated that HO isoforms were localized in the endoplasmic reticulum, HO-1 was also found to be compartmentalized in nuclei, mitochondria, and caveolae [33]. Bioinformatic analysis demonstrates that HO-1 has a nuclear import amino acidic sequence. In the primary astroglial culture system, HO-1 can be induced by excitotoxic injury with concomitant nuclear translocation [34]. HO-1 can translocate into the nucleus under hypoxia or stress conditions with a reduction in HO activity [35]. This nuclear localization of HO-1 may activate certain oxidant-response transcription factors, such as activator protein-1 and NF-κB, and then promote cytoprotection, including cellular proliferation and DNA repair [36,37]. Other subcellular localizations described for HO-1 include mitochondria and caveolae. The localization of HO-1 protein in mitochondria plays an important role in the modulation of mitochondrial heme protein turnover and in protection against pathophysiological condition such as neurodegenerative diseases [38]. Finally, HO-1 has been observed in caveolae exerting a vesicular transport function and involved in receptor signal transduction [39].

Aside from the intracellular compartments, the presence of HO-1 in extracellular compartments and biological fluids has been evaluated. Serum HO-1 is increased in Alzheimer’s disease and exhibits a positive correlation with cognition impairment grade [40]. Schipper HM et al. showed that HO-1 is decreased in the cerebrospinal fluid of patients with AD [41]. HO-1 is increased in the cerebrospinal fluid of children after severe traumatic brain injury [42,43] and patients with Fisher Grade III aneurysmal subarachnoid hemorrhage [44]. These observations demonstrate that HO isoforms, especially HO-1, may influence the physiological functions of the brain via non-canonical effects and serve as a possible biomarker for these diseases. However, there are limited data to this end, and the possible release mechanism(s) of HO-1 in serum or the cerebrospinal fluid remain to be elucidated. In summary, HO-1 is considered to be a survival factor in the brain in response to stress-induced ROS increase.

### 2.3. HO-1 in Brain Physiology

HO-1 is the inducible isoform of heme oxygenase. Under normal conditions, the expression of HO-1 protein in the brain is low and restricted to localized parts [45]. However, rat model studies indicated that HO-1 mRNA is detectable at high levels in the hippocampus and cerebellum, indicating a cellular reserve of HO-1 for quick protein synthesis [46]. Although HO-1 is present at low levels in most mammalian tissues, it can be upregulated by a number of stimuli [47]. In order to study the effect of the enzyme on human physiology, a gene-knockout animal model or a study of human HO-1 deficiency would represent a good way to delineate the role of this protein in various organs.

The important role of HO-1 has been demonstrated in studies on *HMOX1* knockout (HO-1-null) mice. The first HO-1-null mice were established by Poss and Tonegawa in 1997 [48,49]. HO-1-null mice are characterized as an animal model of human hemochromatosis and present with several similar symptoms, such as splenomegaly, iron deposition in tissues, fibrosis and hepatic injury, a mobility decrease, and premature mortality. As compared to cells from wild-type embryos, the embryonic fibroblasts from HO-1-null mice exhibited an increased production of free radicals and reduced survival rate under exposure to several oxidants [49]. Moreover, the first human case of HO-1 deficiency was described in a 6-year-old boy in 1999 by Yachie et al. [50,51] and the second in 2009 by Radhakrishnan et al. [52]. The symptoms in these cases were far more severe under oxidative stress than in HO-1 knockout mice (comparison data in [52] and [50]). The symptoms observed in HO-1-deficiency patients include abnormalities of the fibrinolysis/coagulation system, enhanced systemic inflammation, iron-deficiency anemia/intravascular hemolysis, nephropathy, vascular endothelial injury, and developmental failure [52]. These data demonstrate that HO-1 deficiency is associated with many dangerous side effects, and this accounts for the early death of patients with severe HO-1 deficiency. Interestingly, amyloid deposition, the central neuropathological abnormality in AD and in many neurodegenerative diseases [53], was also observed in severe HO-1 deficiency. These observations indicate that the HO-1 signal plays a crucial anti-oxidative and anti-inflammatory function in modulating human physiology. Thus, how to modulate the HO-1 activity in the brain and what the role of HO-1 is in the development of neurodegenerative diseases are critical to brain pathophysiology.

## 3. Epigenetic Regulation of HO-1

### 3.1. Polymorphisms of HO-1 Promoter

Since HO-1 is an inducible isoform of the HO system, the epigenetic regulations need to be discussed. To date, there are three important polymorphisms of the *HMOX1* promoter to have been identified, including a (GT)n dinucleotide length polymorphism and two single-nucleotide polymorphisms, G(−1135)A and T(−413)A [54].

The current data demonstrate that the lengths of the (GT)n repeat sequence in the HO-1 gene promoter could range from 12 to 40 [55], where <25 (GT)n repeats increase the transcriptional activity of *HMOX1* as compared with >25 (GT)n repeats [56]. In studies on lymphoblastic cell lines, *HMOX1* expression was enhanced in cells with shorter repeats concomitant with higher HO-1 activity upon oxidative stress resulting in oxidant-induced apoptosis as compared with cells with longer (GT)n repeats [56]. However, the length of the HO-1 (GT)n promoter varies between different ethnic groups [56]. These observations indicate that the repeat of the (GT)n sequence has a modulating effect on the transcriptional activity of *HMOX1*.

Two single-nucleotide polymorphisms, G(−1135)A and T(−413)A, were discovered using the PCR method. They were then confirmed by transfection into bovine aortic endothelial cells [57]. The major allele of T(−413)A-(GT)_30_ polymorphism was shown to have greater promoter activity as compared with another major allele, A(−143)A-(GT)_23_ [57]. However, the function of the G(−1135)A polymorphism is still not known [58]. Some evidence indicates that the promoter polymorphisms of *HMOX1* are associated with certain clinical diseases, such as emphysema in smokers [59], hypertension in women [57], and renal transplantation [60,61]. However, microsatellite polymorphism data do not indicate any association between *HMOX1* promoter polymorphism and the development of AD and PD [62].

### 3.2. Post-Transcriptional Modification by MicroRNA (miRNA)

MiRNAs are a large pool of small non-coding RNAs (approximately 21–23 nucleotides long) for post-transcriptional regulation in animals and plants [63]. In mammals, miRNAs are known to control approximately 30% of all protein-coding genes by mediating mRNA degradation or translational repression. Several studies show that miRNAs are involved in the development of neurological diseases, such as miR-142-5p [64], miR-146a, miR-155 [65], and miR-144 [66]. Furthermore, HO-1 targeting miRNAs were also documented in in vitro and in vivo studies, as is summarized in Table 1.

Senescence-accelerated mouse-prone 8 (SAMP8) is an ideal AD model which is characterized by several behavior disorders, including cognitive function impairment and Aβ accumulation with increased oxidative stress [67]. In SAMP8 mice, the expression of Hmox1 is increased concomitant with decreased expression of miR-873-5p, and a luciferase reporter assay indicated that miR-873-5p directly targets the Hmox1 gene [68]. Through an in silico analysis of the 3′UTR sequence, miR-377 and miR-217 were shown to be the miRNA candidates of HMOX1. Co-transfection of miR-377 and miR-217 into mammalian cells decreases the expression of HMOX1-3′UTR luciferase reporter activity as compared with controls [69,70], indicating that miR-217 together with miR-377 could modulate HMOX1 expression. Moreover, in a rodent model, HO-1 was shown to be a specific target of miR-155, which promoted T-cell-driven inflammation [71]. In C. carpio, miR-155 and miR-181a are involved in regulating immune-cytotoxicity of cadmium by targeting HO-1 [72]. However, miR-218-5p was demonstrated to have a cytotoxic effect in septic mice resulting from HO-1 downregulation [73]. Furthermore, the replication of porcine reproductive and respiratory syndrome virus may also be enhanced by miR-24-3p through the downregulation of HO-1 expression [74].

**Table 1:** HO-1 targeting miRNAs and their functions.

miRNA	Species	Functions	Reference
miR-24-3p	Porcine	Promote Porcine Reproductive and Respiratory Syndrome Virus Replication	[74]
miR-155	Carp Rodent	Regulate the immunotoxicity of cadmium in the kidneys Promote T-cell-driven inflammation	[71,72]
miR-181a	Carp	Regulate the immunotoxicity of cadmium in the kidneys	[72]
miR-217 & miR-377	Human	Cytotoxic effect by HO-1 downregulation	[69,70]
miR-218-5p	Mouse	Cytotoxic effect in septic mice by HO-1 downregulation	[73]
miR-873-5p	Mouse	Cytoprotective effect for suppression of neuron cell apoptosis	[68]

### 3.3. Post-Translational Modification

HO-1 was first identified with one consensus sequence for Akt phosphorylation at Ser^188^ in an isotopic ^32^P-labeling assay [75]. In HEK293T cells, the phosphorylation level of HO-1 is increased with Akt1 activation. Furthermore, phosphorylated HO-1(S188D) protein showed a 1.7-fold increase in activity as compared with wild-type HO-1 [75]. Salinasa et al. first reported that the protein kinase Akt plays a vital role in the regulation of HO-1 activity. Interestingly, in AD subjects, HO-1 protein activity was significantly increased in the hippocampus concomitant with an increase in Ser-residue phosphorylation [76]. This Ser-residue phosphorylation seems to be correlated with oxidative post-translational modifications in the hippocampus, indicating that HO-1 has a role in the development of AD. These studies demonstrate that HO-1 activity could be modulated by phosphorylation through oxidative post-translational modification.

## 4. The Redox-Mediated HO-1 Induction in the CNS

Aside from the epigenetic regulation of *HMOX1*, the promoter region of *HMOX1* consists of one proximal and two or more distal enhancers [47]. The promoter region has different binding sequences for many transcription factors, such as nuclear factor-erythroid factor 2-related factor 2 (Nrf2), nuclear factor kappa B (NF-κB), hypoxia-inducible factor 1 (HIF-1), activator protein 1 (AP-1), etc. As described in various studies, Nrf2 plays an important role in redox homeostasis of the brain and nervous system [77]. However, the most well-known transactivation of *HMOX1* by oxidative stress in the brain is the binding of transcription factor Nrf2 to cis-acting antioxidant response element (ARE) enhancers [78].

Nrf2 is a redox-related transcription factor and is responsible for the activation of several antioxidant enzymes [79]. Nrf2 is retained in the cytoplasm under a basal condition by its negative regulator Keap1 (Kelch-like erythroid cell-derived protein with CNC homology-associated protein 1) to undergo ubiquitination and proteasomal degradation [80]. However, under oxidative stress, Keap-1 is modified and releases Nrf2 into the nucleus, binding to the ARE sequences before activating *HMOX1* expression [81]. As a result of Nrf2 binding to the ARE sequence in the presence of small Maf (sMaf) proteins in the nucleus, the BTB domain and CNC homolog 1 (Bach1) protein are other negative regulators of HO-1 activation [82]. Bach1 is a heme-binding protein and can dimerize with sMafs, which prevents the binding of Nrf2 to ARE sequences [83]. These studies demonstrate that, under stress condition such as an increase in the heme group or oxidative stress, Keap1 and Bach1 are modified and then improve Nrf2-sMafs dimerization, thus promoting binding to ARE sequences and activating *HMOX1* expression.

Interestingly, aside from Nrf2-dependent signaling, previous studies demonstrated that there is another pathway to induce HO-1 expression in brain astrocytes [84]. Activation of ERK/NF-κB and JNK/c-Jun cascades as the result of a Nox/ROS-dependent event enhances c-Fos/AP-1 activity and is essential for HO-1 upregulation and the activation induced by bradykinin (BK) in brain astrocytes. Moreover, ROS-dependent Nrf2 activation also contributes to HO-1 induction by BK in astrocytes [84]. Furthermore, the high-glucose-derived oxidative stress-dependent HO-1 expression from astrocytes contributes to the neuronal apoptosis, and the induction of HO-1 is mediated by MAPK-mediated NF-κB and AP-1 cascades [85]. However, these studies suggest that the upregulation of HO-1 may have neurotoxic effects in addition to its protective effects in the CNS [86].

## 5. The Beneficial and Detrimental Role of HO-1 Induction in Neurodegenerative Disorders

As HO-1 is an inducible enzyme in the nervous system’s response to damage, the effect of HO-1 induction in neurodegenerative diseases needs to be further elucidated. Human neurodegenerative disorders are complicated and vary with many factors, such as onset age, sex predilections, neurological and behavioral symptoms, etc. Among these differences, the most common risk for neurodegenerative disorders is age-related factors. There are many general neuropathological features in neurodegenerative diseases, such as oxidative damage resulting from modification to biological molecules, excessive deposition of non-transferrin-bound iron, and macroautophagy in affected neural regions. The evidence indicates that the number of HO-1-immunoreactive neuron cells increases with age, indicating that HO-1 plays a Janus-faced role in brain physiology. Here, we use AD and PD to illustrate how HO-1 is involved in the pathogenesis of CNS degenerative disorders.

An extensive literature attests to the protective roles of HO-1 in the nervous system under various oxidative stress conditions. AD is a neurodegenerative disease characterized by a set of hallmark brain lesions, such as aggregation of the hyperphosphorylated MAPT (tau) protein in neurofibrillary tangles, β-amyloid aggregation in fibrillary plaques, and a neuro-inflammatory response [87]. HO-1 overexpression reduced tau expression and β-amyloid toxicity in neuroblastoma cells and increase neuronal survival in cell and rat models [88,89,90,91]. Furthermore, the protective role of HO-1 in AD brains may also be related to the ability to convert heme, which has a pro-oxidant effect, into its degradation products, which have an antioxidant effect, creating a suitable redox microenvironment [92]. PD is a common neurodegenerative disorder with an unknown etiology. The typical clinical features of PD involve bradykinesia, resting tremor, and rigidity, and in the later stages, postural instability. The development of this movement disorder is due to the loss of dopaminergic neurons in the substantia nigra pars compacta with intracellular aggregation of α-synuclein and the formation of Lewy bodies and Lewy neurites [93]. In vivo and in vitro research indicates that HO-1 induction increases α-synuclein proteasomal degradation [94], prevents dopaminergic neuronal death by enhancing neurotrophic factor generation [95,96], and promotes the antioxidant response [97]. However, these types of HO-1 induction seem to be highly associated with the Nrf2/ARE signal, demonstrating the impact of the Nrf2/HO-1 pathway on neuroprotection function.

Although previously proposed as a protective effect in AD and PD development, the physiological feature of HO-1 in these neurodegenerative diseases is still under debate. Interestingly, HO-1 is overexpressed in the brain of AD patients by co-localization with neurons, astrocytes, ependymal, corpora amylacea, neurofibrillary tangles, and senile plaques [98,99]. It is also overexpressed in nigral astroglia and in dopaminergic neuronal Lewy bodies of the PD brain [98,99]. HO-1 overexpression in astroglia promotes the oxidation of cholesterol to oxysterols in humans and increases oxysterol levels with a decrease in the intracellular cholesterol content in rat [100,101]. The status of plasma HO-1/biliverdin reductase-A has been proposed as a potential biomarker to detect the earliest stages of AD [102]. Moreover, high glucose-induced HO-1 expression is mediated through the NF-κB and AP-1 pathways in brain astrocytes [85]. All these data support the detrimental role of HO-1 induction in the development of neurodegenerative diseases, especially via an astrocytes-mediated event.

Since HO-1 induction plays a dual role in neuropathogenesis, the function of HO-1 in neuronal cells and in astrocytes, oligodendrocytes, and microglia needs to be considered at the stage of neurodegenerative disorders. Indeed, the role of HO-1 expression is highly complicated and not fully elucidated. However, whether HO-1 induction plays cytoprotective or cytotoxic effect in neuropathogenesis may be related to different signaling pathways [103]. That is to say, Nrf2-dependent activation of HO-1 exerts a cytoprotective effect, in which AP-1- or NF-κB-induced HO-1 activation seems to exert cytotoxic effects in the CNS.

## 6. Herbal Medicine Induces HO-1 Expression

Since HO-1 induction via Nrf-2 pathway in brain plays main functions for preventing brain damage, there are several HO-1 inducers/modulators for therapy or potential therapeutic functions, such as herbal medicine, hemin [104], edavarone [105], cobalt protoporphirin [9], and adenoviral vector transferring system [106]. However, due to the adjuvant functions of herbal compounds and easy supplement from food, we here only summarized those herb medicines as HO-1 inducers. The herbal medicine data for this review were obtained from the ClinicalTrials.gov (accessed on 29 March 2022) database and include resveratrol, curcumin, coenzyme Q10, sulforaphane, niacin, propolis, atorvastatin, and dimethyl fumarate, which could be involved in HO-1 induction.

### 6.1. Resveratrol

Resveratrol, 3,5,4′-trihydroxy-trans-stilbene, belongs to the phytoalexin family and is produced by red grapes, red cherries, peanuts, and berries. It is popular as a dietary supplement and the studies demonstrate that it has various health-promoting properties including anti-inflammatory, antioxidant, and neuroprotective effects [107]. However, resveratrol exhibits poor bioavailability due to its instability and poor lipophilic properties. Resveratrol exerts therapeutic effects on neurodegenerative diseases. Resveratrol treatment was shown to improve autonomic dysfunction and motor function in a rat model of spinal cord injury [108]. Furthermore, resveratrol improved BBB integrity as a result of anti-oxidation by upregulating the Nrf2/HO-1 and PI3K/Akt signaling pathways and anti-inflammation by attenuating the activity of NF-κB and JNK/MAPK signals [109,110]. In addition, its major neuroprotective function in AD results from its anti-protein aggregation and anti-amyloidogenesis properties through the abolishment of neurofibrillary tau protein tangles or Aβ protein formation and deposition; thus, it is able to improve brain cognition function [111,112]. Resveratrol could protect dopaminergic SH-SY5Y neuron cells from rotenone-induced cell death in a HO-1-dependent autophagy manner [113]. Although resveratrol’s protective function for cognition is mediated by AMPK/SIRT1 signaling, the network between those anti-inflammatory responses needs to be further elucidated. Hence, resveratrol can improve cognitive function in patients with neurodegenerative diseases and further clinical trials are required to delineate its neuroprotective role.

### 6.2. Curcumin

Curcumin, 1,7-bis(4-hydroxy-3-methoxyphenyl)-1,6-heptadiene-3,5-dione, is a pigment and active polyphenol found in turmeric (in the ginger family) [114]. Curcumin is the main compound contributing to the biological functions of turmeric, and it is common as a food supplement. Curcumin has many biological functions, such as antioxidant, anti-inflammatory, anti-diabetic, anti-microbial, and neuroprotective properties, due to its ability to pass through the BBB effectively. Curcumin is denoted as “Generally Recognized As Safe” by the US Food and Drug Administration [115] with good safety and tolerability in clinical trials [116,117]. The neuroprotection properties of curcumin are mediated through improving the Nrf2/HO-1 pathway (antioxidant response) and by inhibiting the NF-κB, TLR4/RAGE, and MAPKs (ERK, p38, and JNK) signaling pathways (anti-inflammatory response) in microglial and astrocytes [118]. As a result of the anti-amyloidogenesis and anti-protein aggregation/misfolding properties, curcumin has demonstrated positive effects against neurodegenerative disorders, especially AD [119]. However, like resveratrol, curcumin exhibits poor bioavailability, and increasing curcumin’s bioavailability should be a focus of future research. Further trials are required concerning curcumin’s neuroprotective functions against other neurodegenerative diseases.

### 6.3. Coenzyme Q10 (CoQ10)

Coenzyme Q10 (CoQ10) plays the role of an electron acceptor in energy metabolism to produce ATP. It is found in food sources such as organ meat, fatty fish, and broccoli. As a result of its lipophilic capacity, CoQ10 also acts as a potent antioxidant and possesses a wide range of therapeutic effects. Moreover, it is effective against various neurodegenerative diseases as it passes through the BBB [120]. Its potent neuroprotective properties are mediated by activating the endogenous antioxidant system via the Nrf2/HO-1 signaling pathway and attenuating the NF-κB-mediated inflammatory pathway to protect the dopaminergic neuron system. In addition, ubiquinol-10, the reduced form of CoQ10, was shown to be safe and improve PD by lowering total Unified Parkinson’s Disease Rating Scale (UPDRS) scores. CoQ10 supplementation was shown to improve PD symptoms in various clinical studies and it has potential as a complementary therapy [121].

### 6.4. Sulforaphane

Sulforaphane, 1-isothiocyanato-4-(methylsulfinyl) butane, is an aliphatic isothiocyanate found in glucoraphanin in cruciferous vegetables such as broccoli, cauliflower, and cabbage [122]. Sulforaphane is characterized as having antioxidant, anti-inflammatory, and anti-apoptosis properties. It was shown to inhibit oxidative stress via the Keap1/Nrf2/ARE pathway by modulating the expression of GSH peroxidase 1, NQO-1, HO-1, and gamma-glutamylcysteine synthetase [123]. Furthermore, sulforaphane can also reduce neuronal damage upon microglial activation and inhibit the expression of inflammatory mediators, such as TNF-α, IL-1β, inducible nitric oxide synthetase (iNOS), cyclooxygenase-2 (COX-2) and macrophage migration inhibitory factor [124,125,126,127,128,129,130]. As a result of its good oral bioavailability and its ease of crossing through the BBB [131], an increasing number of studies demonstrate the efficacy of sulforaphane as a therapeutic strategy in neurodegenerative disease [132]. Therefore, sulforaphane could be used as a supplement for treating neurodegenerative diseases.

### 6.5. Niacin

The brain is the most cholesterol-rich organ, and cholesterol content may regulate synaptic function and neuronal cell plasticity [133]. Current studies demonstrate that there is a significant correlation between total cholesterol and pathologically defined AD [134,135]. Niacin is the most potent agent for increasing HDL cholesterol, inhibiting inflammation, and promoting vascular remodeling. Niacin inhibits vascular inflammation via the induction of HO-1 by Nrf2/p38 MAPK signaling [136]. However, whether niacin could be used as a therapy for AD needs further elucidated.

### 6.6. Propolis

Propolis, a mixture of bee saliva, beeswax, and substances from plants and trees, is a natural product found in beehives that possesses a therapeutic role in PD treatment. Several lines of evidence indicate that flavonoids in propolis demonstrate neuroprotective properties in dopaminergic neurons through the inhibition of oxidative stress [137]. The flavonoids in propolis include caffeic acid phenethyl ester, chrysin (5,7-dihydroxyflavone), and pinocembrin, which easily pass through the BBB and exert antioxidant and anti-inflammatory activities [138]. Pinocembrin treatment was shown to induce the expression of the HO-1 by Nrf2/ARE pathway, significantly reducing MPP^+^-induced neurotoxicity, ROS production, and the rate of apoptosis and neuron cell death [139,140]. Furthermore, caffeic acid phenethyl ester also exerts protective effects in nigral dopaminergic neurons from 6-hydroxydopamine hemiparkinsonian mice through HO-1 and brain-derived neurotrophic factor signals.

### 6.7. Atorvastatin

Statin is a common therapeutic strategy for hypercholesterolaemia; however, as previous discussed in the section describing niacin, a significant link between cholesterol and the development of AD has been observed, thus statin therapy might be of benefit for AD pathogenesis [141]. Cholesterol-lowering statins have several biological functions, such as anti-inflammatory, antioxidative, anti-thrombogenic, and immunological effects. Among these statins, atorvastatin has been demonstrated to have benefits in terms of improving AD outcomes. It significantly improves depressive symptoms and cognitive functions at 6 months, and improves cognitive function and psychiatric symptoms at 12 months [141,142]. Furthermore, in a dog preclinical AD model, atorvastatin treatment induced HO-1 expression, providing neuroprotection by modulating oxidative stress [143].

### 6.8. Dimethyl Fumarate

Among fumaric acid esters, dimethyl fumarate, the methyl ester of fumaric acid, has effective pharmacological functions and exerts anti-inflammatory and antioxidant properties [144]. Dimethyl fumarate is able to cross the BBB and exhibit beneficial effects in the brain via differing mechanisms [145]. Dimethyl fumarate plays the role of an Nrf2 inducer and exerts a neuroprotective role in several neurodegenerative diseases, such as AD, PD, and Huntington’s disease [146].

Although the herbal medicines mentioned above may provide neuroprotective effects through the modulation of HO-1 expression, the bioavailability and lipophilic properties of these medicine must be explored in order to assess their stability and how affective they are at permeating the blood brain barrier (BBB). Nanocarriers represent an interesting solution as a potential drug delivery candidate for passing through the BBB [147]. Thus, how these medicines can be utilized as treatments or preventatives for the development of neurodegenerative disorders is an important issue, and further clinical trials are required.

## 7. Conclusions

**Figure 2:**
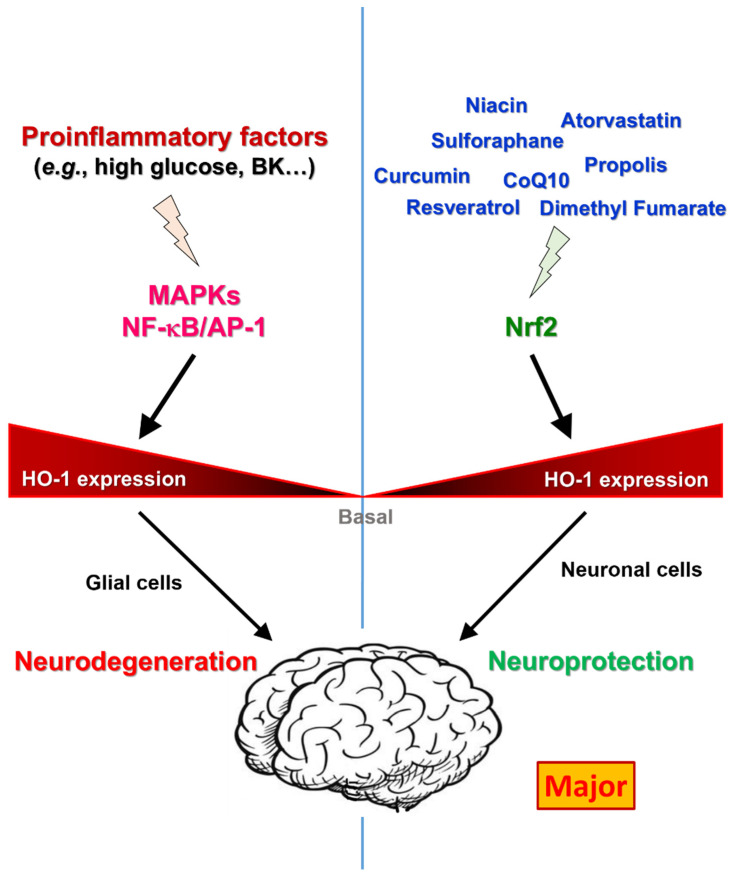
Schematic representation of the HO-1 regulation and its role in the procession of neurodegeneration and neuroprotection. In neurodegeneration, proinflammatory factors induce HO-1 expression via MAPKs, NF-κB, and AP-1 in glial cells. However, several herbal medicines induce HO-1 expression via Nrf2-dependent pathway in neuronal cells, indicating the major route of HO-1 induction for neuroprotective function. Thus, in brain, the final impact of upregulated HO-1 is depend on the stimulatory factors, the activated signaling pathways, and the stimulated cell-type.

Although HO-1 has been observed to have cytoprotective and cytotoxic effects in the development of neurodegenerative diseases, HO-1 activity needs to be maintained in a well-defined reaction which involves the generation and degradation of heme. Heme metabolism or Nrf2-mediated HO-1 induction in neuronal cells exerts protective effects against many stressors; however, excessive activation of HO-1 by the NF-κB/AP-1 pathway may produce cytopathic effects, depending on the complex of cell–cell interactions or the type of brain tissue. Furthermore, the dysregulation of the heme degradation pathway may alter iron metabolism, leading to neurodegeneration in neurons and glial cells. Neurodegenerative diseases are complex and multifactorial diseases, and interventions should be considered during the long preclinical phase. The currently available drugs have symptomatic effects, with the majority playing the role of an Nrf2 inducer and increasing the expression of HO-1 in order to modulate oxidative stress, as shown in Figure 2. Taken together, these reports indicate that HO-1 induction, especially through Nrf2 pathway, may alleviate the brain damage and plays important therapeutic functions in neurodegenerative diseases. However, the detail mechanism of HO-1 on the cytotoxic effect of glial cells needs to be further elucidated.
